# Pemetrexed Induced Life-threatening Anaphylaxis

**DOI:** 10.7759/cureus.5514

**Published:** 2019-08-29

**Authors:** Venkat Rajasurya, Bimatshu Pyakuryal, Kulothungan Gunasekaran, Vijaykumar Sekar, Prajwal Dhakal

**Affiliations:** 1 Pulmonary Critical Care, Novant Health, Winston Salem, USA; 2 Internal Medicine, Nepal Medical College, Kathmandu, NPL; 3 Pulmonary Critical Care, Yale New Haven Health at Bridgeport Hospital, Bridgeport, USA; 4 Internal Medicine, Bassett Medical Center, Cooperstown, USA; 5 Hematology and Oncology, University of Nebraska Medical Center, Omaha, USA

**Keywords:** anaphylaxis, pemetrexed, chemotherapy, lung cancer, treatment side effects, respiratory failure, acute respiratory failure, drug allergy, adverse drug reactions

## Abstract

Non-small cell lung cancer (NSCLC) is the leading cause of cancer-related death. The American Society for Clinical Oncology (ASCO) recommends platinum based regimens as the first-line of treatment for NSCLC. Pemetrexed, an antifolate agent, has been approved by the ASCO for the treatment of advanced non-squamous NSCLC and has been shown to be efficient for first-line, maintenance and second- or third-line treatment in this subgroup. It is administered intravenously over 10 minutes and is usually well tolerated with a very few side effects. There have been a few cases of anaphylaxis reported with pemetrexed use and most of the patients presented only with cutaneous manifestations. We present a patient with stage IV adenocarcinoma of the lung who developed a severe life threatening anaphylactic reaction requiring ventilatory support after administration of pemetrexed.

## Introduction

NSCLC accounts for almost 85% of all lung cancer cases and nearly half of all newly diagnosed lung cancer patients have stage IV cancer [1]. Improving the survival and reducing the disease-related adverse events are the main goals of treating these patients. Cytotoxic combination chemotherapy is the first-line therapy for stage IV NSCLC [2]. The ASCO guidelines states that treatment for a patient with a performance status of 0 or 1 is a regimen of a platinum (cisplatin or carboplatin) plus paclitaxel, gemcitabine, docetaxel, vinorelbine, irinotecan or pemetrexed [3]. Pemetrexed is an antifolate agent and has become one of the most frequently prescribed chemotherapeutic agents for advanced nonsquamous NSCLC which is usually well tolerated, with very few adverse effects [4]. We present a patient with stage IV adenocarcinoma of the lung who developed severe life threatening anaphylactic shock and acute hypoxic respiratory failure after she received intravenous infusion of pemetrexed.

## Case presentation

A 53 year old female with stage IV lung adenocarcinoma presented to the emergency department with acute onset dyspnea that started suddenly when she was receiving intravenous infusion of chemotherapeutic agent pemetrexed. Prior to the infusion, she did not have any respiratory complaints and had a normal chest exam with clear breath sounds to auscultation. She had a history of severe COPD and uses 2 liters supplemental oxygen continuously. She smokes a pack of cigarettes every day and has accumulated 40 pack-year smoking. She was allergic to levofloxacin and doxycycline. She had previously received 5 cycles of carboplatin and pemetrexed and was responding well to the treatment. Upon infusion of the sixth cycle of pemetrexed, she developed severe shortness of breath with wheezing. Her vital signs were oxygen saturation of 80% on room air, blood pressure of 90/60 mm Hg and a heart rate of 110 bpm. Her lung exam revealed reduced airflow in both the lungs with wheezing. She was minimally responsive to painful stimuli and was in severe respiratory distress. She was started on normal saline intravenous fluids. She was emergently intubated using Macintosh 3 direct laryngoscope by EMS personnel in the outpatient infusion center. Prior to intubation, she was given 0.5 mg intramuscular epinephrine. She did not have angioedema on oral examination and was transferred to the intensive care unit. Laboratory studies were normal. Chest X ray did not show any acute infiltrates, except for a stable chronic right sided loculated pleural effusion and CT angiogram did not reveal pulmonary embolism. Her hypotension resolved with intravenous fluid boluses and she did not require vasopressors. She was also given dexamethasone, diphenhydramine, ranitidine and nebulized albuterol. Her ICU course was uneventful and she was extubated after 48 hours. She was discharged home on a oral prednisone taper. Pemetrexed was discontinued and she was started on atezolizumab. Because of the interval disease progression, she was transitioned to bevacizumab and docetaxel. 

## Discussion

Systemic therapy improves the survival and quality of life of patients with advanced stage NSCLC. Several new therapeutic regimens have emerged for advanced NSCLC. Pemetrexed is a folate antimetabolite and it is chemically related to methotrexate and folate in the thymidylate synthetase inhibitors group. It acts by disrupting folate-dependent metabolic processes essential for cell replication [5]. It is approved by the FDA to be used in combination with cisplatin to treat malignant pleural mesothelioma in non-resectable and non-surgical patients. It is also approved for the treatment of nonsquamous NSCLC [2]. Pemetrexed is associated with a low incidence of adverse events including myelosuppression, neutropenic infection, renal failure and interstitial pneumonitis. Very few cases of mild hypersensitivity reactions have been reported including cutaneous adverse reactions [6]. Anaphylaxis is an acute, potentially life-threatening, multi-system syndrome caused by the sudden release of mast cell mediators into the systemic circulation. Common signs of anaphylaxis include flushing, urticaria, hypotension, increased ventilatory pressure, and in severe cases, inability to ventilate because of severe bronchospasm [7]. Our patient developed this severe anaphylactic reaction during the sixth cycle of pemetrexed.

There have been very few cases of hypersensitivity reactions to pemetrexed that have been reported in the literature. These patients presented with cutaneous manifestations like acute generalized exanthematous pustulosis, urticarial vasculitis, epidermal necrolysis and radiation recall dermatitis [8]. Although these reactions happened immediately after pemetrexed administration, most of them were due to direct cytotoxicity following the cell cycle arrest rather than true immune-mediated reaction [8]. So far, there has been only four patients reported with Type I or immediate hypersensitivity reactions who presented with non life-threatening symptoms that improved with intravenous fluids, steroids and antihistamine administration [9-12]. In contrast our patient had life-threatening anaphylaxis requiring endotracheal intubation and ventilator support. Since pemetrexed is frequently used in combination with other chemotherapeutic drugs agents, it is possible that immediate hypersensitivity reactions to pemetrexed is underestimated. Adverse effects of pemetrexed are listed in Table 1.

**Table 1:** Adverse effects of Pemetrexed Source: Lexicomp

Organ system	Adverse effects
Central nervous system	Fatigue (18% to 34%), Neuropathy (sensory: 9%; motor: ≤5%)
Dermatologic	Desquamation (≤14%), Skin rash (≤14%); Pruritus (7%), Alopecia (6%), Erythema multiforme (≤5%)
Gastrointestinal	Nausea (12% to 31%), Anorexia (19% to 22%), Vomiting (6% to 16%), Stomatitis (≤15%), Diarrhea (5% to 13%), Constipation (6%), Abdominal pain (1% to <5%)
Hematologic & oncologic	Anemia (15% to 19%), Neutropenia (6% to 11%), Thrombocytopenia (8%), Febrile neutropenia (<5%)
Respiratory	Pharyngitis (≤15%)
Cardiovascular	Edema (5%)
Hepatic	Increased serum alanine aminotransferase (8% to 10%), Increased serum aspartate aminotransferase (7% to 8%)
Others	Hypersensitivity reaction (<5%), Infection (1% to 5%), sepsis (1%), Conjunctivitis (≤5%), Increased lacrimation (1% to <5%), Fever (8%)

## Conclusions

Pemetrexed is a potent chemotherapeutic agent that is being increasingly used as a frontline agent in NSCLC. Anaphylaxis is an unanticipated severe allergic reaction and it is important to recognize this rare but fatal adverse drug reaction when pemetrexed is administered. Anaphylaxis can occur anytime although the patient tolerated the same treatment well before without any complications due to delayed sensitization. Careful monitoring, prompt recognition and appropriate supportive care is essential to prevent mortality due to anaphylactic reaction due to pemetrexed.
